# The Gulf Stream

**Published:** 1881-01

**Authors:** 


### THE GULF STREAM.

There is a river in the ocean. In the severest drouths it never fails, and in the mightiest flood it never overflows. Its banks and its bottom are of cold water, while its current is of warm. The Gulf of Mexico is its fountain, and its mouth is in the Arctic seas. It is the Gulf Stream. There is in the world no other so majestic a flow of water. Its current is more swift than the Mississippi or the Amazon, and its volume more than 1,900 times greater. Its waters so far as the Carolina coasts are indigo blue. They are so distinctly marked that the common sea-water can be traced with the eye. Often one-half the vessel may be seen floating in the Gulf stream waters while the other half is in the common water of the sea, so sharp is the line and want of affinity between these waters, and such, too, the reluctance, so to speak, on the part of those of the Gulf Stream to mingle with the common waters of the sea. In addition to these, there is another peculiar fact. The fishermen on the coast of Norway are supplied with wood from the tropics by the Gulf stream. Think of the Arctic fishermen burning upon their hearths the palms of Hayti, the mahogany of Honduras and the precious woods of the Amazon and the Orinoco.

---

EUREKA! exclaimed Socrates, as he sat down on the business end of a tack that he had been looking for.

KEROSENE will soften boots or shoes which have been hardened by water, and render them as pliable as when new. It will also make tin kettles as bright as when new. Saturate a woolen rag and rub with it. Stains may also be removed from clean varnished furniture with kerosene.

FOR CLEANING KID GLOVES.—Get one quart of deodorized benzine, one drachm of sulphuric ether, one drachm of chloroform, two drachms of alcohol. Pour a little of this into a clean bowl, and wash the gloves in it as you would wash anything. After the dirt is nearly out, rinse in more of the clean fluid. Usually one rinsing is enough, but if the gloves were very much soiled, rinse the second time. If the gloves are of cheap kid it is best to dry them on the hand, but a nice glove, after having been rubbed with a soft cloth to smooth out wrinkles, may be hung on a line to dry. This preparation is an excellent thing to keep in the house, not only for cleaning gloves, but for taking out grease spots from clothing and carpets, and for sponging coat collars and felt hats.

